# Evaluating Environmentally Sustainable Development Based on the PSR Framework and Variable Weigh Analytic Hierarchy Process

**DOI:** 10.3390/ijerph18062836

**Published:** 2021-03-10

**Authors:** Fan Wang, Yao Lu, Jin Li, Juan Ni

**Affiliations:** 1 School of Accounting, Zhejiang Gongshang University, Hangzhou 310018, China; wangfan1031@mail.zjgsu.edu.cn (F.W.); luyaochongya@sina.com (Y.L.); 2 School of Management and E-Business, Modern Business Research Center, Key Research Institute, Zhejiang Gongshang University, Hangzhou 310018, China; jinli@mail.zjgsu.edu.cn; 3 School of Public Finance and Taxation, Zhongnan University of Economics and Law, Wuhan 430073, China

**Keywords:** environmentally sustainable development, pressure-state-response framework, variable weigh analytic hierarchy process

## Abstract

Environmentally sustainable development is a multidimensional concept that emphasizes the integration of economy, society and environment within a region and the realization of dynamic balance. How to objectively environmentally sustainable development has been a major concern for scholars and policy makers. To address this problem effectively, we first obtain the indicators of environmentally sustainable development based on the pressure-state-response (PSR) framework. Then, we introduce variable weight factors in the traditional analytic hierarchy process (AHP), so that the weights assigned by experts to sustainable development indicators can change with time or space. In this way, we propose a new and improved weight distribution method called variable weigh analytic hierarchy process. Finally, we employ indicators of environmentally sustainable development based on PSR and variable weigh analytic hierarchy process to evaluate the sustainable development of cities in a case country. Our study found that: (1) indicators of environmentally sustainable development should consist of three parts: pressure indicators of environmentally sustainable development, state indicators of environmentally sustainable development, and response indicators of sustainable development; (2) with the variable weigh analytic hierarchy process, our ranking hierarchy process can handle dynamic changes among indicators better than the traditional AHP method and better reflect the true states of indicators.

## 1. Introduction

Environmentally Sustainable Development is a multi-dimensional concept, which emphasizes integration and striking a dynamic balance between economic, social and environmental aspects in a region, to ensure intergenerational equity [1]. From September 25 to 27 of 2015 at the World Summit on Environmentally Sustainable Development, 193 member states of the United Nations officially adopted the document “Transforming our World: The 2030 Agenda for Sustainable Development”. According to the conclusion of the Global Environmental Outlook 5 [2], the achievement of environmental sustainability is not satisfactory [3]. Despite some progress in environmental protection, the problems of steady deterioration of the ecological environment, unsustainable use of the natural resources, and climate change are still serious.

How to evaluate environmentally sustainable development more objectively has been a major concern for scholars. However, there is no unified evaluation model in the world. In order to establish a widely applicable model of environmentally sustainable development, this article first constructed indicators of environmentally sustainable development based on the pressure-state-response (PSR) framework. At the same time, this paper introduces variable weight factors into the environmentally sustainable development evaluation. Finally, using the indicators of environmentally sustainable development based on PSR and the variable weigh analytic hierarchy process constructed in this article, we conducted an urban environmentally sustainable development evaluation on the case country, i.e., China. The main contributions of this paper are: (1) Using PSR theory to construct an indicator system for environmental sustainability to provide a basis for scholars’ follow-up research; (2) Introducing some variable weight factors into the AHP model to construct a variable weigh analytic hierarchy process to make The factor weight changes with the factor value, which makes up for the relatively fixed defect of the factor weight of the AHP model; (3) These methods are applied to the case to verify the feasibility of the method.

The rest of the paper is arranged as follows. First, we identify environmental sustainability indicators based on PSR. The indicator layer is constructed according to the “Pressure-State-Response” framework. Second, the variable weight factor is introduced into the AHP model, and the variable weight analysis hierarchy process is established. Third, we use the analytic hierarchy process of indicators and variable weights to analyze data related to sustainable environmental development in China for three consecutive years and the corresponding variable weights and constant weights. Fourth, through the analysis and calculation of constant weights and variable weights, we draw conclusions on environmentally sustainable development.

## 2. Literature Review

The framework of pressure, state and response (PSR) has been used in the environmental assessment since it was proposed by Friend and Rapport in 1979 [4]. Now the Organization for Economic Co-operation and Development Organization for Economic Cooperation and Development (OECD) [5,6] and the United Nations Environment Program (UNEP) [7] use it to study the framework of environmental issues. Pressure indicators of environmentally sustainable development (P) refer to the pressure and state change of human economic and social activities on the ecological environment. State indicators of environmentally sustainable development (S) refer to the current and past ecological environment status and conditions, reflecting the natural ecological environment, human health and living environment. Response indicators of environmentally sustainable development (R) refer to a series of actions taken by humans to reduce the pressure on the ecological environment or change the ecological environment.

In order to further extend the above models, this paper introduces variable weight factors [8] into the environmentally sustainable development evaluation. Specifically, it refers to the addition of variable weight factors in the traditional AHP, so that the weights of experts change with time or time and space. That is, the core of variable weight theory is that the change of the state value of the factor will cause the weight of the factor itself to change, thus adapting to the responses of different decision making units, a new, more scientific and reasonable weight distribution method is formed-variable weigh analytic hierarchy process.

To evaluate the present situation of Environmentally Sustainable Development, we need to comprehensively consider the coordinated development of the resources, environment, and economy. Commonly-used methods in the literature include the analytic hierarchy process (AHP) [9,10], ecological footprint [11], material flow analysis [12,13], energy analysis [14,15], model of data envelopment analysis (DEA) [16,17], and fuzzy comprehensive evaluation [18,19]. However, the factor weight of the analytic hierarchy process is relatively fixed, so that it cannot handle the dynamic changes that may exist among the indicators [20]. Fuzzy comprehensive evaluation only allows experts to give multiple fuzzy scores. It does not substantially improve the traditional analytic hierarchy process but adds a lot of burden to the scoring experts [21]. The ecological footprint, material flow analysis, energy analysis, and DEA do not involve the subjective judgment of experts, so that the evaluation of sustainable development does not reflect the important environmental issues considered by humans.

It’s worth noting that the variable weight introduced in this paper can also be applied in project evaluation and empowerment in most countries in the world, and the environmental sustainability model proposed provides a guidance to lots of countries. At the same time, each country can adapt the corresponding evaluation items to its own geographical location, natural environmental conditions and other factors. For example, island countries such as New Zealand and Japan pay more attention to the water environment, and landlocked countries pay more emphasis on the atmospheric environment.

## 3. Pressure-State-Response Framework

### 3.1. Introduction to PSR: Materials and Methods

The environmentally sustainable development index system is composed of three categories of indicators: pressure, state and response [22]. To answer “what happened”, “why it happened” and “how to do”, the PSR framework constructs the index with the logic of “pressure effects—state changing—problem solution”. “What happened” can be answered by the state indicators which indicate the physical changes (or biological changes) or trends in nature and the state of the corresponding socioeconomic development trend. These state indicators are used to measure the environmental quality or environmental status of environmentally sustainable development, especially the changes caused by human activities and the impact on humans. The pressure indicators of environmental changes caused by human activities are to answer “Why it happened”. For instance, some human behaviors such as exploiting or over-using the resources, discharging pollutants or waste gas into environment and intervening in environment lead to depletion of resources and deterioration of environmental quality, and the pressure indicators can measure the pressure on the environment. The response indicators of human countermeasures for environmental problems answer the questions of “what has been done” and “what should be done”, showing that the society makes efforts to solve the environmental problems and measure the status of implementation of environmental policies for environmentally sustainable development. Starting from the pressure of sustainable environment, with the logic of the PSR framework, the improvement of the environmental state and the environmental governance will enhance the effect of sustainable evaluation and assist the environmentally sustainable development [23].

### 3.2. Indicators of Environmentally Sustainable Development Based on PSR

Based on the theory of PSR, the pressure indicators of environmentally sustainable development (P) refer to the impact of human economic and social activities on the ecological environment. Humans’ demand for environmental resources destroys the ecological balance. The production and management activities produce waste water, waste gas, and residue waste which pollute the ecological environment. The state indicators of environmentally sustainable development (S) refer to the current and past ecological environment status and situation, which reflect the natural ecological environment, human health and living environment. The response indicators of environmentally sustainable development (R) are a series of actions humans have made to reduce the pressure on the ecological environment or change the state of the ecological environment, such as the enforcement and supervision of environmental law, environmental legislation and improvement of it, environmental protection, and strengthening public awareness.

Combining the connotations of PSR in the environmentally sustainable development model above, we use all indicators that may reflect environmental sustainability. We select the pressure, state, and response indicators of environmentally sustainable development, as shown in Table 1.

## 4. Analytic Hierarchy Process Based on Variable Weigh

### 4.1. Introduction to Analytic Hierarchy Process Based on Variable Weigh

Although after undergoing many changes and iterations in various fields, and providing tremendous contributions to the development of society and economy, AHP, of which factor weight fixed in essence, cannot cope with the extraordinary situation where the relative order is chaotic due to the interaction of elements in complex systems. The main reason is that the basic AHP cannot deal with possible dynamic problems between criteria and index. The analytic hierarchy process based on variable weigh method can effectively cope with the fixed weight of traditional AHP. That is, based on determining the weight by the experts, the weight given by the expert is corrected by real data, which makes the changed weight to be closer to the real result. The core content of variable weight theory is that, as the state value of the factor changes, the weight of the factor changes so as to adapt to the requirements of different decision-making on the response factor.

The theory of variable weight can effectively deal with the fixed weight of traditional AHP. Introducing the theory of variable weight based on determining weight, a new, scientific and reasonable weight assignment method, analytic hierarchy process based on variable weigh method, is formed. Incentive variable weight focuses on the stimulation of key factors, which is more sensitive to the increase of high-level single-factor state value and is unresponsive to the reduction of low-level single-factor state value. On the contrary, penalized variable weight puts emphasis on the balance between factors, so it is more sensitive to the reduction of low-level single-factor state value, and is unresponsive to the increase of high-level single-factor state value. With the characteristics of both incentive and punitive, mixed variable weight incents evaluation factors at a certain level and punishes them at a certain low level.

### 4.2. The Method of Analytic Hierarchy Process Based on Variable Weigh

Compared with the traditional AHP, the analytic hierarchy process based on variable weigh further magnifies the impact of high-risk factors, so in the process of evaluation, it is helpful to reveal the true impact of risk factors on the evaluation system. The calculation procedure of analytic hierarchy process based on variable weigh is as follow:

Firstly, non-dimensionalize the raw data. Due to the large dimensional differences between different indicators, we need to standardize the raw data first. Because the extreme value method has the characteristics of high applicability and no requirement on the quantity and distribution of data, the non-dimensionalized data are all between 0-1, and has the characteristic of relative number. Following the extreme value method in Gregory and Jackson [24], we use it as the method of non-dimensionalize, which is shown in Equations (1) and (2) below.

Maximum value:

(1) $$x_{ij}=\frac{\mu_{ij}}{\mu_{j}{}^{\max}}$$

Minimal value:

(2) $$x_{ij}=\frac{\mu_{j}{}^{\max}}{\mu_{ij}}$$

where $\mu_{ij}$ refers to the raw data; $\mu_{j}^{\max}$ refers to the maximum value in the *j*-th indicator; $\mu_{j}^{\min}$ refers to the minimum value in the *j*-th indicator; and $x_{ij}$ refers to the non-dimensionalized data.

Secondly, determine the constant weight vector. For indicators of index-level, the weights are determined by the method of analytic hierarchy process. That is, the constant weight vectors of the indicators of the index layer are obtained from the questionnaires issued to relevant experts and scholars. For the indicators of the criterion layer, we use the results of score of experts’ questionnaires to determine the weight of environmental sustainability pressure, environmental sustainability state and environmental sustainability response. The constant weight vector of the index layer is denoted as $W_{i}=(w_{i1},w_{i2},\ldots ,w_{ij}),(i=1,2,3;j=1,2,\ldots ,m)$, and the definition of the constant weight vector of the criterion layer is denoted as $D=(d_{1},d_{2},d_{3})$.

Thirdly, determine the state variable weight vector and the variable weight vector. According to Table 1, the index system of environmental sustainability evaluation is divided into two layers: criterion layer and index layer. The factor state value of the index layer is denoted as $x_{ij}(i=1,2,3;j=1,2,..,m)$, and the factor state vector is denoted as $X_{i}=(x_{i1},x_{i2},\ldots ,x_{ij})$; the evaluation value of criterion-level indicators is denoted as $y_{i}(i=1,2,3)$, and the factor state vector is denoted as $Y=(y_{1},y_{2},y_{3})$.

For the indicators of each index layer in the same criterion layer, once there is a big difference between the indicator values, it indicates that the balance of the state value of each factor is poor, which means that there is a problem in the Environmentally Sustainable Development of the environment of the city. Therefore, we choose a penalized state variable weight vector to modify this result, specifically shown in Formulas (3) and (4). δ is the parameter which is greater than 0, is the state variable weight vector of the index layer, and $W_{i}(X)$ is the variable weight $S_{i}(X)$ vector of the index layer.

(3) $$S_{ij}(x_{i})=e^{-\delta (x_{ij}-\bar{x_{i}})}$$

(4) $$W_{i}(X)=\frac{\left(w_{i1}S_{i1}(x_{i}),\ldots ,w_{im}S_{im}(x_{i})\right)}{\sum_{j=1}^{m}w_{ij}S_{ij}(x_{i})}$$

For the criterion layer, we should also carry out variable weight processing, but the principle of the criterion layer is different from that of the index layer, shown in Formulas (5) and (6). The Formula (5) is the state variable weight vector (y1) of the criterion layer of the pressure for environmental sustainability ($S\left(y_{1}\right)$). Since the pressure of environmental sustainability refers to the destruction of the environment by human beings, we adopt the penalized state variable weight vector. If it is lower than a penalty level α, a penalty will be given. On the contrary, if it is greater than or equal to a penalty level α, neither penalty nor incentive will be given. That means, when the pressure of environmental sustainability is below a certain threshold, the negative impact is considered to exceed the acceptable range.

(5) $$S(y_{1})=\left\{\begin{array}{c} e^{-(\alpha -y_{1})}\\ 1 \end{array}\right.\begin{array}{c} y_{1}<\alpha \\ y_{1}\geq \alpha \end{array}$$

For the state of environmental sustainability (y2) and the response of environmental sustainability (y3), the values reflect the current environmental sustainability of the city and how the local governments cope with the adverse effects of human activities on the environmental sustainability. As different local governments show different emphasis on environmental sustainability, some cities have better state of environmental sustainability and response of environmental sustainability, while others are worse off. Therefore, we cannot use the model of penalized state variable weight vector or the model of incentives for state variable weight vector, but a model of hybrid state variable weight vector. Formula (6) is the state variable weight vector of the criterion layer of the state and response of the environmental sustainability $S\left(y_{i}\right),i=2,3$. When $y_{i}$ is lower than a certain constant level of penalty α(0 < α < 1), α penalty will be given; when it is higher than or equal to a certain constant level of incentive b (0 < b < 1), an incentive will be given. When it is between the penalty level α and the incentive level b, neither penalty nor incentive will be given.

(6) $$S(y_{i})=\left\{\begin{array}{c} e^{-(\alpha -y_{i})}\\ 1\\ e^{\left(y_{i}-b\right)} \end{array}\right.\begin{array}{c} y_{i}<\alpha \\ \alpha \leq y_{i}<b\\ y_{i}\geq b \end{array}i=2,3$$

According to the state variable weight vector of the criterion layer $S\left(y_{i}\right),i=1,2,3$ which is calculated with Formulas (5) and (6), we calculate the variable weight vector as follows:

(7) $$W(Y)=\frac{\left(d_{1}S(y_{1}),\ldots ,d_{3}S(y_{3})\right)}{\sum_{i=1}^{3}d_{i}S_{i}(y)}$$

Fourthly, calculate the score of integrated evaluation. In terms of the factor state vector Xi and the constant weight vectors Wi, D, we calculate the integrated score of constant weight z, as shown in Formulas (8) and (9).

(8) $$y_{i}=\sum_{j=1}^{m}\left(x_{ij}\times W_{i}\right)\begin{array}{cc} & i=1,2,3 \end{array}$$

(9) $$z=\sum_{i=1}^{3}(y_{i}\times D)$$

According to the factor state vector *X_i_* and the variable weight vector of the index layer $W_{i}(X)$, the factor state vector can be calculated; then according to the factor state vector and the variable weight vector of the criterion layer W(Y), the integrated score of variable weight *z* is calculated, as shown in Formulas (10) and (11).

(10) $$y_{i}=\sum_{j=1}^{m}\left(x_{ij}\times W_{ij}(X)\right)\;i\;=\;1,2,3$$

(11) $$z=\sum_{i=1}^{3}\left(y_{i}\times W_{i}(Y)\right)$$

### 4.3. Sustainability Evaluation System Weight Table

When the consistency ratio C.R < 0.1, the matrix can be considered as a satisfactory consistency matrix. That is, its normalized feature vector can be used as a constant weight vector. The consistency check is a necessary step, and the weights of all levels should meet the consistency requirements. In order to obtain the weight of this article, we distribute seven questionnaires to scholars who study environmental management; officials from government environmental protection agencies; corporate environmental managers, and recovered six valid questionnaires. We chose these representatives to fill out the questionnaires because environmental research scholars, as a third party, can objectively evaluate environmental pressures, governance, and other factors while government environmental protection agency officials, acting as supervisors, have an inescapable responsibility for environmental response. On the other hand, as a polluter, corporate environmental managers have different scores for environmental pollution evaluation and the other two topics in the economic interests.

In fact, we surveyed seven experts, each of whom filled out a questionnaire that included criteria level, P, S, and R, four tables that allowed two-to-two comparisons. Each expert assigned weights to the indicators in the questionnaire. Among them, the six experts’ weights passed the consistency test (the six experts’ C.R. average results are shown in Table 2), but the weight of expert 7 did not pass the consistency test, so we removed the weighting from expert 7. Finally, we use the first 6 expert weighted geometric mean as the constant weight of this index system.

Through calculation, all conform to the consistency test, as shown in Table 2.

Based on the confirmation of the questionnaire, the final standing weight result is obtained. The six experts who passed the consistency check gave the weighted geometric mean as Constant Weights (due to space constraints, the tables are not specified here.), thus, it can be seen from Table 3, experts and scholars pay more attention to environmental sustainability measures, so they give more weights on them. This shows that they believe that environmental sustainability responses should be more worthy of the government’s attention than environmental sustainability conditions and pressures. The importance of the response of continuous development is greater than the other two standards. In terms of indicators, the weight of pressure of water environment is lower than that of pressure of atmospheric environment. In terms of the intuitive perception of experts, scholars and ordinary people, the pressure of the atmospheric environment is more important than the pressure of the water environment. Similarly, this situation also exists between state of atmospheric environment and state of solid waste, and there is also a certain weight gap between the two. However, in the indicator layer of Response to environmental sustainability, the weights of the three are not much different, which means that in the eyes of government workers and environmental researchers, the degree of governance of the water environment, atmospheric environment, and solid environment is about the same.

## 5. Sample and Indicator

### 5.1. Sample

The “Environmental Protection Law of the People’s Republic of China” was issued in 2015. As a result, the disclosed data has a gap in the calculation caliber around 2015, which is not comparable. Therefore, the samples used in the empirical analysis are the environmental sustainability data of 30 province-level regions in China from 2016 to 2018, with a total of 90 samples. The data come from “China Statistical Yearbook (CSY)”, “China Environmental Yearbook (CEY)” and “China Environmental Statistics Yearbook (CESY)”.

### 5.2. Indicator of Description

After averaging the statistics of the actual data of 30 province-level regions in China (Beijing, Tianjin, HeBei, LiaoNing, ShangHai, JiangSu, Zhejiang, Fujian, Shandong, Guangdong, Hainan, Shanxi, Jilin, Heilongjiang, Anhui, Jiangxi, Henan, Hubei, Hunan, Neimenggu, Guangxi, Chongqing, Sichuan, Guizhou, Yunnan, Shanxi, Gansu, Qinghai, Ningxia, Xinjiang), the descriptive statistical analysis results of all variables are obtained, as shown in Table 4. Variables with less dispersion include VANWD, TVNPWD, SDXE%IGDP, NOXE%IGDP, SDE%IGDP, and IEGIEGI, of which standard deviations are all less than 10. The variable with the largest degree of dispersion is IIWT, which reaches 245,996.40. However, in order to reasonably evaluate the magnitude of the fluctuations of variables, it is necessary to resort to the coefficient of variation, which incorporates the level of the variable into the measurement of variable variation. As shown in Table 4, the variables with the highest fluctuations are SVHW, GHW, and DVGISW, and their fluctuations between variable values are the greater. The least fluctuating variable is RUST, and its fluctuation is relatively stable. At the same time, from the perspective of the change of the overall average, most of the pressure indicators of environmentally sustainable development in 2016–2018 are gradually declining, and the state indicators of environmentally sustainable development are getting better, but the response indicators of environmentally sustainable development are uncertain.

### 5.3. Analysis of the Results

We separately extract the principal components of the pressure indicators of Environmentally Sustainable Development, the state indicators of Environmentally Sustainable Development, and the response indicators of Environmentally Sustainable Development. The original data are standardized and the correlation coefficient matrix is obtained. Bartlett’s test and Kaiser–Meyer–Olkin (KMO) were used for the validity of the scale [25,26]. The sphericity values of Bartlett’s test are 1180.496, 349.502, 273.735, and all significance are 0.000, so it can be considered that the correlation coefficient matrix is not the identity matrix [27]. The value of KMO statistics is between (0–1). 0 indicates that there is no relationship between the original variables, and 1 indicates that there is an important relationship [24]. The KMO test values in this paper are all greater than 0.6, so the data are suitable for factor analysis, as shown in Table 5.

**Figure 1:**
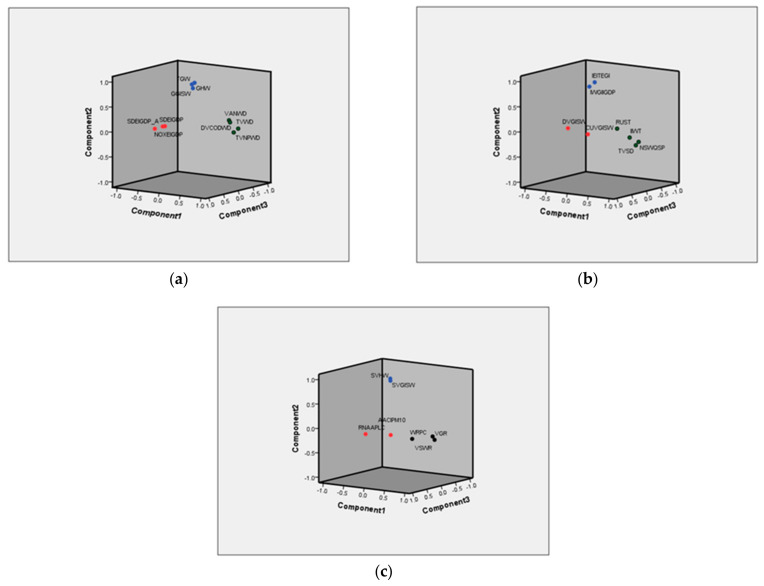
Principal Component Analysis Diagram of Environmentally Sustainable Development; (**a**) Principal Component Analysis Diagram of Pressure of Environmentally Sustainable Development; (**b**) Principal Component Analysis Diagram of State of Environmentally Sustainable Development; (**c**); Principal Component Analysis Diagram of Response to Environmentally Sustainable Development.

Subsequently, this paper will find the characteristic root and factor loading matrix. Rotating the data to maximize variance, in terms of the principle that the eigenvalue is greater than 1, the principal component analysis of environmental pressure, environmental state and environmental governance extracts three principal components F1, F2, and F3 respectively. If the cumulative contribution rate of the extracted factors reaches more than 80%, it is believed that the newly extracted principal component can basically reflect most of the information of the original indicator data, that is, the analysis obtained from the extracted principal component of the original variable is effective, and it is enough to describe the environmental pressure, state, and response. See Figure 1.

It can be seen from the rotated factor loading matrix that the component 1 of the pressure of Environmentally Sustainable Development is mainly determined by the four indicators of TVWD, DVCODWD, VANWD, and TVNPWD, which represents the pressure of wastewater discharge on the environment and can be summarized as the factors of the water environment pressure. Component 2 is mainly determined by the three indicators of GGISW, GHW, and TGW, which represent the pressure of solid waste on the environment and can be summarized as the factors of solid waste pressure. Component 3 is mainly determined by three indicators: SDE%IGDP, NOXE%IGDP, and SDXE%IGDP, which means the pressure produced by each GDP on atmospheric environment can be summarized as the factors of atmospheric environmental pressure. In component 1 of the state of environmentally sustainable development, the three indicators of WRPC, VSWR, and VGR account for a large proportion, which can be summarized as factors of water environment state. Component 2 is mainly determined by two indicators: GGISW and GHW, which can be summarized as factors of solid waste state. Component 3 is mainly determined by two indicators: RNAAPLC and AACIPM_10_, which can be summarized as the factors of atmospheric environmental state. Component 1 of the response of environmentally sustainable development is mainly determined by four indicators: TVSD, IIWT, RUST and NSWQSP, which represent the treatment of sewage and wastewater and can be summarized as factors of water environment treatment. Component 2 is mainly composed of IWGI%IGDP and IEGIEGI, which represent the government’s emphasis on atmospheric environmental governance and can be summarized as atmospheric environmental governance factor. Component 3 is mainly determined by the CUVGISW and DVGISW, which represent the utilization and disposal capacity of solid waste and can be summarized as factors of solid waste response. As shown in Table 6.

### 5.4. Discussions

According to the state variable weight vector calculation Formulas (3), (4), and (6), and the variable weight vector calculation Formulas (4) and (7), as well as the environmental data of the 30 provinces and cities in China from 2016 to 2018, the principal components were extracted. The average variable weight vectors are obtained respectively, as shown in Table 7 below.

In Table 7, the maximum weight of the pressure of environmental sustainability criterion layer is 0.232 in Shanghai, and the lowest is 0.053 in Shandong. The difference between the variable weights of the two is relatively large. A certain item at the criterion level, such as the environmental sustainability discovery status project, is more important, so the variable weight is more important than other provinces and cities. In terms of State of environmental sustainability, Guangdong’s power to change rights is greater than that of other provinces and cities, which means that Guangzhou has invested more energy in this aspect and has given more attention. In terms of response to environmental sustainability, Guangdong’s variable weight is significantly lower than Shandong, and there is a large difference between the two. This phenomenon shows that, to some extent, Shandong pays more attention to response to environmental sustainability.

Similarly, Shandong has a large difference in the weights of the three criterion levels. Shandong’s weight in response to environmental sustainability is eight times that of the other two criterion levels and is more active in response. Except for several provinces and cities such as Qinghai and Guizhou, most provinces and cities place variable weights on Response to environmental sustainability. Regions such as the Yunnan-Guizhou Plateau have given the State of environmental sustainability a greater proportion due to factors such as fragile environment and unstable ecological development. Among the 30 provinces and cities in China, Shanghai has a more even distribution of variable weights. The three variable weights are 0.232, 0.379, and 0.389, which are inseparable from its geographic location and economic development. Shanghai’s economic development is fast, and the pressure for sustainable environmental development is not small. After a series of measures such as environmental remediation, Shanghai’s environmental sustainability has been improved. Therefore, the Shanghai government aims to better maintain the environment.

### 5.5. Sustainable Capacity and Balanced Distribution

According to the above formulas and steps, we average and analyze the results of three years’ constant contingency weights of 30 province-level regions in China.

After averaging the results, comparing to the scores of constant weight results, the scores of variable weight results reduced with different degrees, which shows that some values of state factors are “unbalanced” in both index layer and criterion layer. Environmental assessment includes the pressure of atmospheric environmental, the pressure of water environmental, the state of water environmental and environmental response, of which various internal sub-responsibilities are “disharmonious”. Therefore, they have received a certain degree of “penalty”, which leads to the comprehensive evaluation value of variable weight being low. If being “penalized”, it means some matters in a certain area are not handled well, which makes the composite scores drop China’s provinces of Shandong, Yunnan, and Fujian rank first, third, and fourth, respectively, in both the scores of constant weight and variable weight, which indicates that these provinces are not changed after varied weight processing, probably because the better natural environment is, the relatively higher score is, especially for Yunnan province. Yunnan Province is rich in natural resources. It is known as the “vegetable kingdom”, “animal kingdom”, “non-ferrous metal kingdom”, and “hometown of medicinal materials”, and has gained the reputation of “the south of colorful clouds”. Because the weather in Yunnan province is mild, the utilization rate of coal is lower than that of cold northern cities, so the air is fresh and the environment is good. However, Dianchi Lake in Yunnan province is slightly polluted, and the main pollution indicators are demand volume of chemical oxygen and total phosphorus. Among the 10 monitored water quality points, Grade IV accounts for 60.0% (China divides water quality into five categories. Grade I: good water quality. Groundwa-ter only needs to be disinfected, and surface water can be used for drinking after simple purification (such as filtration) and disinfection. Grade II: the water quality is slightly polluted. After conventional purification treatment (such as flocculation, sedimentation, filtration, disinfection, etc.), the water quality can reach to daily drinking. Grade III: suita-ble for the secondary protection area of the centralized drinking water source, general fish protection area and swimming area. Grade V: suitable for general industrial protection areas and recreational water areas that are not in direct contact with the human body. Grade IV: suitable for agricultural water areas and general landscape requirements. Water bodies that exceed the five types of water quality standards basically have no use function.), Grade V accounts for 40.0%, and there are no Grade I, II, III, or inferior Grade V. Through the treatment, comparing to those indicators of 2017, the proportion of water quality points of Grade IV increased by 60.0%, and the percentage of water quality points of inferior Grade V decreased by 60.0%. The water quality of Shandong province has continued to improve for 16 consecutive years, with a water quality ratio of 46.3%, and the air has continued to improve for six years (data source: Shandong Province 2018 Environmental Bulletin). A good natural ecological environment and favorable governance have laid the foundation for the above provinces.

The rankings of Henan province, Guangxi province, and Anhui province are in a below average level in the constant weight results, but the results of variable weight integrated assessment method rank second, fifth, and seventh respectively, of which the scores are higher than other provinces and cities. The advantages of these provinces’ natural environment are not prominent. The Henan Provincial Environmental Bulletin in 2019 showed that the national ecological environment index (EI, environment index.) (China divides water quality into five categories. Grade I: good water quality. Groundwater only needs to be disinfected, and surface water can be used for drinking after simple purification (such as filtration) and disinfection. Grade II: the water quality is slightly polluted. After conventional purification treatment (such as flocculation, sedimentation, filtration, disinfection, etc.), the water quality can reach to daily drinking. Grade III: suitable for the secondary protection area of the centralized drinking water source, general fish protection area and swimming area. Grade V: suitable for general industrial protection areas and recreational water areas that are not in direct contact with the human body. Grade IV: suitable for agricultural water areas and general landscape requirements. Water bodies that exceed the five types of water quality standards basically have no use function.) value was 51.3, and the ecological quality was not good. The count area with excellent and good ecological quality accounted for 44.7% of the total land area, average -quality area accounted for 22.7%, and fair and poor accounted for 32.6%. However, Henan province payed attention to environmental response, and served the overall situation. In accordance with the provincial deployment of environmental pollution prevention and control, it completed 428 sets of the emergency control and the automatic monitoring facilities of exhaust gas of peak-shift production enterprises, and 714 sets of construction and networking of total nitrogen automatic monitoring facilities in 2018. At the same time, it guided and urged relevant cities and counties to complete the installation pilot tasks of VOCS online monitoring facilities and video monitoring facilities. Combining the needs of the accomplishment of tough tasks and the management and control of weather in the day of heavy pollution, Henan province actively monitored pollutant emissions and exceeding standards of enterprises, regularly prepared various special monitoring reports, and put forward relevant countermeasures and suggestions, which provided effective data support for the implementation of the whole province’s tough task.

Table 8 shows the ranking of score of constant weight and variable weight of 30 provincial-level regions in China in 2016–2018. In the first column of Table 8, the following notations are defined. ASCWA: average score of constant weight; RCW: ranking of constant weight; ASVW: average score of variable weight; RVW: ranking of variable weight. It can be seen from the table that, compared with the ranking of constant weight, the ranking of variable weight of each province has been changed, indicating that the contingent analytic hierarchy process uses real data to correct the permanent power score and obtains a more realistic variable ranking. This ranking of variable weight shows that Beijing (RCW is 4, RVW is 15), Fujian (RCW is 3, RVW is 4), Guangdong (RCW is 2, RVW is 6), and Jiangsu (RCW is 4, RVW is 15) are all lower than the Ranking of constant weight, especially Tianjin, whose score of constant weight ranked first, but the variable weight score ranked around 20th. Economic development may be achieved at the cost of the ecological environment, but economic development cannot be at the expense of the ecological environment, and environmentally sustainable development is limited by the environmental carrying capacity.

## 6. Conclusions

Environmentally sustainable development refers to the coordinated development of economy, society, resources, and environmental protection which are all involved in the same inseparable system. This system not only helps people to achieve the goal of economic development, but also protects the natural resources which humans can depend on for survival. However, many regions and countries have paid insufficient attention to the ecological and environmental issues. Moreover, there are companies which even sacrifice the natural environment for profit maximization.

To help accurately assess the environmentally sustainable development level of cities in a region or country, this article first constructed indicators of environmentally sustainable development based on PSR. At the same time, in order to overcome the shortcoming of traditional evaluation method, this paper proposes a new weight distribution method on the basis of variable weight theory—analytic hierarchy process based on variable weight which can adjust the weight with the change of the value of factor state, so as to deal with the environmental response factors which can affect environmentally sustainable development in a balanced way and make the evaluation result established on a more objective and reasonable basis. Finally, through the case analysis of environmental sustainability assessment of 30 province-level regions in China, the characteristic of flexibility and effectiveness of this method is proved, and a certain idea for the research of index system of sustainable environmental assessment is provided. The research in this article can continue to be used in various dynamic evaluation items, such as evaluating some quality-related items. At the same time, the variable weight analysis method is also very useful when assigning weights, which overcomes the shortcoming that the weight of constant weight does not change with the state. The variable weight method can also be applied to other countries in the world, and the PSR indicator system can be extended based to the situation of different countries around the world.

This study shows that: (1) indicators of environmentally sustainable development should include three parts: pressure indicators of environmentally sustainable development (P), state indicators of environmentally sustainable development (S), and response indicators of environmentally sustainable development (R). (2) When the variable weigh analytic hierarchy process is applied, the ranking of the environmentally sustainable development capability of the urban environment has changed, indicating that the variable weigh analytic hierarchy process is better than the traditional AHP method to deal with possible dynamic changes among indicators. (3) This ranking of score of variable weight shows that ranking of variable weight of Beijing, Fujian, Guangdong, Jiangsu, and Tianjin are all lower than the ranking of constant weight. It may be due to the economic development is at the cost of ecological environment. We believe that economic development cannot be at the expense of the ecological environment, and environmentally sustainable development is limited by the environmental carrying capacity.

However, it is worthwhile to note that although the indicators of environmentally sustainable development established in this article are applicable to most countries, if there are specific indicators in a certain region or country, the specific conditions of these countries and regions should also be considered, and the application needs to be adjusted. For example, Japan has a lot of marine resources. If this method is applied to Japan, indicators related to the marine environment should be added to the indicator system.

## Figures and Tables

**Table 1:** Variable Descriptions and Measurement Unit.

Classification	Indicator Names	Abbreviation	Measurement Scale
P	Total volume of wastewater discharge	TVWD	10,000 tons
	Demand volume of chemical oxygen in wastewater discharge	DVCODWD	10,000 tons
	Volume of ammonia- in wastewater discharge	VANWD	10,000 tons
	Total volume of nitrogen-phosphorus in wastewater discharge	TVNPWD	10,000 tons
	Sulfur dioxide emissions per unit of industrial GDP ^1^	SDXE%IGDP	%
	Nitrogen oxide emissions per unit of industrial GDP	NOXE%IGDP	%
	Smoke and dust emissions per unit of industrial GDP	SDE%IGDP	%
	Generation of general industrial solid waste	GGISW	10,000 tons
	Generation of hazardous waste	GHW	10,000 tons
	Total generation of waste	TGW	10,000 tons
S	Water resources per capita	WRPC	cubic meters per capita
	Volume of surface water resources	VSWR	hundred million cubic meters
	Volume of groundwater resources	VGR	hundred million cubic meters
	Ratio of non-attainment air in prefecture-level city	RNAAPLC	%
	Annual average concentration of inhalable PM_10_^2^	AACIPM10	
	Stock volume of general industrial solid waste	SVGISW	10,000 tons
	Stock volume of hazardous waste	SVHW	10,000 tons
	Total volume of sewage disposal	TVSD	million cubic meters
R	Investment in industrial wastewater treatment	IIWT	10,000 Yuan
	Number of surface water quality section points	NSWQSP	point location
	Rate of urban sewage treatment	RUST	%
	Industrial waste gas investment accounts for industrial GDP	IWGI%IGDP	%
	Comprehensive utilization volume of general industrial solid waste	CUVGISW	10,000 tons
	Disposal volume of general industrial solid waste	DVGISW	10,000 tons
	Industrial exhaust gas investment accounts for environmental governance investment	IEGIEGI	%

^1^ per unit of industrial Gross Domestic Product (GDP); ^2^ Inhalable particles (abbreviation: PM_10_), refers to particles with an aerodynamic equivalent diameter ≤10 microns, called inhalable particles, also called PM_10_.

**Table 2:** Consistency Test.

Inspection Index	Criterion Layer 1	First Level Indicator 1	First Level Indicator 2	First Level Indicator 3
λ=	3.007	3.000	3.003	3.003
CI=	0.003	0.000	0.001	0.002
RI=	0.580	0.580	0.580	0.580
CR=	0.006	0.000	0.002	0.003

λ: The largest characteristic root λ of the *n*-th order reciprocal matrix, and when λ = *n*, it is a uniform matrix; CI: consistency index; RI: random consistency index; CR: consistency ratio.

**Table 3:** Constant Weights.

Indicator Name	Constant Weight	Indicator Name	Constant Weight
Pressure of environmental sustainability	0.27	Pressure of atmospheric environment	0.41
		Pressure of Water environment	0.29
		Pressure of solid waste	0.30
State of environmental sustainability	0.31	State of atmospheric environment	0.39
		State of water environment	0.31
		State of solid waste	0.29
Response to environmental sustainability	0.43	Response to water environment	0.29
		Response to atmospheric environment	0.37
		Response to solid waste	0.34

**Table 4:** Descriptive Statistics of Variables.

Variable	Descriptive Statistics					Trend of Overall Average Change		
	Mean	SD	Coef.	Min.	Max.	2015	2016	2017
TVWD	238,239.87	188,238.36	0.79	23,663	938,261	244,911.43	236,827	232,981.17
DVCODWD	47.6	37.02	0.78	5.8	175.8	74.02	34.79	33.98
VANWD	5.67	3.97	0.7	0.6	20	7.65	4.72	4.64
TVNPWD	10.76	10.98	1.02	0.9	75.8	17.17	7.52	7.6
SDXE%IGDP	0.01	0.01	0.99	0	0	0.01	0.01	0.01
NOXE%IGDP	0.01	0.01	0.79	0	0	0.01	0.01	0.01
SDE%IGDP	0.01	0.01	1	0	0	0.01	0.01	0.01
GGISW	5187.89	7180.05	1.38	0	35,372	10,889.3	2073.57	2600.81
GHW	66.72	117.41	1.76	0	757.5	132.54	38.61	29.03
TGW	5254.62	7225.78	1.38	0	35,430.2	11,021.84	2112.18	2629.84
WRPC	2171.26	2299.26	1.06	83.4	13,188.9	2024.68	2349.44	2139.68
VSWR	807.5	784.74	0.97	7.1	2466	768.25	887.72	766.54
VGR	244.94	188.86	0.77	4.9	762	233.13	260.9	240.79
RNAAPLC	78.35	32.03	0.41	0	100	80.94	77.13	76.97
AACIPM10	85.57	25.19	0.29	37	145.4	91.97	85.73	79.03
SVGISW	2202.53	2968.17	1.35	0	14630	1933.21	2073.57	2600.81
SVHW	31.55	69.19	2.19	0	424.3	27.01	38.61	29.03
TVSD	149,034.6	128,755.85	0.86	11,143	673,323	142,859.1	149,337.5	154,907.2
IIWT	191,916.64	245,996.4	1.28	893	1,143,103.9	38,500.24	270,280.15	266,969.54
NSWQSP	343.56	256.3	0.75	36	1468	333.97	376.23	320.47
RUST	91.85	5.54	0.06	60	97.8	89.96	92.03	93.56
IWGI%IGDP	25.78	28.77	1.12	4.2	180.1	29.16	28.7	19.48
CUVGISW	6267.38	4963.55	0.79	186	19,900	6626.57	6136.1	6039.47
DVGISW	2425.19	3481.47	1.44	3	16,684	2433.1	2182.7	2659.77
IEGIEGI	0.06	0.04	0.64	0	0.2	0.06	0.07	0.05

**Table 5:** Test of KMO and Bartlett.

Index		Pressure of Environmentally Sustainable Development	State of Environmentally Sustainable Development	Response to Environmentally Sustainable Development
Kaiser-Meyer-Olkin metric with sufficient sampling		0.746	0.620	0.609
Bartlett’s sphericity test ^1^	Chi-square approximate	1180.496	349.502	273.735
	Df	45	21	28
	Significance	0.000	0.000	0.000

^1^ Bartlett’s spherical test is a mathematical term. It is used to test the correlation between the variables in the correlation matrix, whether it is a unit matrix, that is, to test whether each variable is independent. Before factor analysis, KMO test and Bartley sphere test are performed first. In factor analysis, if the null hypothesis is rejected, it means that factor analysis can be done. If the null hypothesis is not rejected, it means that these variables may independently provide some information and are not suitable for factor analysis.

**Table 6:** Variables used in the analysis.

Criterion Layer	First Level Indicator	Secondary Indicators
Pressure of environmental sustainability	Pressure of Water environment	TVWD
		DVCODWD
		VANWD
		TVNPWD
	Pressure of solid waste	GGISW
		GHW
		TGW
	Pressure of atmospheric environment	SDXE%IGDP
		NOXE%IGDP
		SDE%IGDP
State of environmental sustainability	State of water environment	WRPC
		VSWR
		VGR
	State of solid waste	GISWS
		VHWS
	State of atmospheric environment	RNAAPLC
		AACIPM10
Response to environmental sustainability	Response to water environment	TVSD
		IIWT
		RUST
		NSWQSP
	Response to solid waste	CUVGISW
		DVGISW
	Response to atmospheric environment	IWGI%IGDP
		IEGIEGI

**Table 7:** Variable weight table.

**Index**	**Beijing**	**Tianjin**	**Hei Bei**	**Liao Ning**	**Shang Hai**	**Jiang Su**	**Zhejiang**	**Fujian**	**Shandong**	**Guangdong**
Pressure of environmental sustainability	0.177	0.173	0.145	0.124	0.232	0.124	0.120	0.108	0.053	0.115
State of environmental sustainability	0.411	0.463	0.276	0.257	0.379	0.319	0.452	0.473	0.110	0.536
Response to environmental sustainability	0.412	0.363	0.579	0.619	0.389	0.557	0.429	0.419	0.837	0.349
	**Hainan**	**Shanxi**	**Jilin**	**Heilongjiang**	**Anhui**	**Jangxi**	**Henan**	**Hubei**	**Hunan**	**Neimeggu**
Pressure of environmental sustainability	0.189	0.157	0.151	0.126	0.112	0.125	0.095	0.130	0.129	0.129
State of environmental sustainability	0.369	0.298	0.388	0.437	0.381	0.475	0.220	0.437	0.453	0.351
Response to environmental sustainability	0.442	0.545	0.461	0.437	0.507	0.400	0.685	0.433	0.418	0.520
	**Guangxi**	**Cong** **qing**	**Sichuan**	**Guizhou**	**Yunnan**	**Shanxi**	**Gansu**	**Qinghai**	**Ningxia**	**Xingjiang**
Pressure of environmental sustainability	0.114	0.153	0.152	0.126	0.102	0.137	0.177	0.142	0.205	0.129
State of environmental sustainability	0.500	0.440	0.463	0.525	0.445	0.317	0.374	0.504	0.308	0.408
Response to environmental sustainability	0.385	0.407	0.385	0.349	0.453	0.546	0.449	0.354	0.486	0.464

**Table 8:** Ranking of score of constant weight and variable weight of 30 provincial-level regions in China in 2016–2018.

**Province**	**Shandong**	**Henan**	**Yunnan**	**Fujian**	**Guangxi**	**Guangdong**	**Anhui**	**Zhejiang**	**Guizhou**	**Jiangxi**
ASCW	2.272	1.936	2.160	2.294	1.960	2.431	1.867	2.135	1.803	1.859
RCW	5	18	8	3	17	2	20	10	25	21
ASVW	2.731	1.977	1.865	1.810	1.763	1.760	1.745	1.693	1.678	1.663
RVW	1	2	3	4	5	6	7	8	9	10
**Province**	**Liaoning**	**Jiangsu**	**Hubei**	**Hunan**	**Beijing**	**Heilongjiang**	**Inner Mongolia**	**Shanxi**	**Xinjiang**	**Tianjin**
ASCW	1.810	2.072	1.857	1.875	2.289	1.712	2.067	1.711	1.812	2.619
RCW	24	12	22	19	4	26	13	27	23	1
ASVW	1.659	1.635	1.611	1.606	1.605	1.591	1.582	1.570	1.556	1.517
RVW	11	12	13	14	15	16	17	18	19	20
**Province**	**Qinghai**	**Chongqing**	**Hebei**	**Jilin**	**Sichuan**	**Shanxi**	**G** **ansu**	**Hainan**	**Ningxia**	**Shanghai**
ASCW	2.193	1.623	2.137	1.674	1.979	2.179	1.517	2.124	2.035	1.996
RCW	6	29	9	28	16	7	30	11	14	15
ASVW	1.516	1.483	1.473	1.466	1.454	1.361	1.238	1.155	1.053	1.026
RVW	21	22	23	24	25	26	27	28	29	30

## Data Availability

Not applicable.
